# Effect of herbal extract *Eurycoma longifolia* (Physta^®^) on female reproductive hormones and bone biochemical markers: an ovariectomised rat model study

**DOI:** 10.1186/s12906-020-2814-z

**Published:** 2020-02-05

**Authors:** Sasikala M. Chinnappan, Annie George, Godavarthi Ashok, Yogendra Kumar Choudhary

**Affiliations:** 1 Biotropics Malaysia Berhad, Lot 21 Jalan U1/19, Section U1Hicom Glenmarie Industrial Park, Shah Alam, Selangor Malaysia; 2 Etica Clinpharm Pvt. Ltd, Raipur, Chhattisgarh 492001 India; 3 Radiant Research Services Pvt. Ltd, 99/A, 8 main, III Phase, Peenya Industrial Area, Bangalore, 560058 India

**Keywords:** *Eurycoma longifolia*, Ovariectomy, Hormonal balance

## Abstract

**Background:**

Each year 1.5 million women experience menopause when menstrual cycles cease resulting from the loss of ovarian function and oestrogen deprivation, a hormone that helps prevent bone loss. This study investigated the effects of *Physta*®, a standardized herbal extract of *Eurycoma longifolia* Jack (PEL), on hormonal balance and parameters associated with hormonal imbalance, namely body and uterus weight and bone biochemical markers relevant in menopausal symptoms.

**Methods:**

Forty-eight Sprague Dawley rats were randomly divided into six groups of eight rats each: (A) Sham operated; control (B) Untreated (ovariectomised (OVX) with vehicle), (C) PEL 100 (OVX + 100 mg/kg body weight (bw)), (D) PEL 300 (OVX + 300 mg/kg bw), (E) PEL 500 (OVX + 500 mg/kg bw) and (F) Positive control, testosterone undecanoate (TU) (OVX+ 10 mg/kg bw). Group A and B received daily oral administrations of the vehicle, Group C-E received daily oral administration of PEL and Group F received testosterone undecanoate intramuscularly weekly. At the end of 8 weeks, serum calcium, phosphate, bone alkaline phosphatase (BALP), osteocalcin, follicle stimulating hormone (FSH), luteinising hormone (LH), oestrogen, progesterone and testosterone were measured, then the animals were sacrificed and uterus was isolated, while weight was recorded in all experimental groups.

**Results:**

Treatment of OVX rats with PEL at a dose of 500 mg/kg showed decreased serum FSH (*P* < 0.001, 4.25 ± 0.22 mIU/ml) and LH (NS, 4.07 ± 0.12 mIU/ml), while there was a significant increase in progesterone (*P* < 0.05, 2.48 ± 0.08 ng/ml) and oestrogen (*P* < 0.05, 11.02 ± 0.13 pg/ml) levels when compared to untreated group. PEL treatment at doses of 100 mg/kg, 300 mg/kg and 500 mg/kg showed a non-significant but increasing trend in serum calcium, phosphate, bone alkaline phosphate and testosterone levels. Ovariectomy resulted in a significant reduction (*P* < 0.001, 238.81 ± 5.39 mg) in uterus weight in the ovariectomised rats, which was alleviated in all PEL treated ovariectomised rats with an increasing trend of uterine weight.

**Conclusion:**

The results suggest that PEL could be protective and beneficial for the management of reproductive hormone and bone markers. Therefore, it could be used to address hormonal imbalances and symptoms associated with menopause.

## Background

Owing to the increased human life expectancy that has resulted from socioeconomic advancements and developments in medical technology, the post-menopausal period now accounts for over one-third of a woman’s lifespan [1]. Menopause is a biological stage in a woman’s life when menstrual cycles cease resulting from the loss of ovarian function and oestrogen deprivation, a hormone that helps prevent bone loss [2, 3]. Women in menopausal transition experience a variety of symptoms such as hot flashes, sweating, anxiety, depression, mood swings, sleep disorders, vaginal dryness and joint pain; all of which are due to the cessation of ovarian oestrogen production [4]. Bone loss also occurs in premenopausal women following ovariectomy (OVX) or treatment with gonadotrophin-releasing hormone agonists [5, 6]. The rapid decline in endogenous oestrogen production that occurs during menopause results in a significant increase in bone turnover, thereby significant bone loss and increased risk for fragility fracture [7]. Ovariectomy is one of the most common surgical operations in women throughout the world, and is associated with an increased rate in bone resorption outweighing the increased rate of bone formation. Consequently, this leads to osteoporosis [8]. Ovarian hormone deficiency is the most important risk factor for post-menopausal osteoporosis [9, 10]. Osteoblasts are cell that synthesise bone, whereas osteoclasts are bone cells that breakdown bone tissue. Stimulation of osteoblast differentiation has been suggested to be an important therapeutic approach for the prevention and treatment of osteoporosis [11].

Bone formation markers (BFM) are by-products of active osteoblasts expressed during different phases of their development and are considered to reflect different aspects of osteoblast function and bone formation [12]. The most widely used BFM are BALP, osteocalcin and the propeptides of type 1 collagen: these markers are measured in serum or plasma [12].

The use of ovariectomy, removal of both ovaries, as a model of oestrogen deficiency-induced hormonal imbalance and bone loss is widespread throughout discovery and pre-clinical translational research [13, 14]. Typically, in this surgical model, bilateral removal of the ovaries occurs in young reproductively competent healthy animals. Experimental interventions occur either at the time of ovariectomy or commence once 17β-estradiol has reached a low to non-detectable level in plasma, which typically occurs within 1–2 weeks [15]. As a discovery strategy, data derived from the ovariectomy animal model has furthered the fundamental understanding of ovarian hormone action in every organ in the body [16].

In women, androgen production takes place in ovary, adrenal and peripheral tissues. Testosterone converted to oestrogen via aromatase activity in the peripheral compartments. Serum testosterone is an important marker of ovarian androgen production [17]. Based on past research other than oestrogen, hormones as progestins, testosterone, dehydroepiandrosterone (DHEA) were used as therapies for managing menopause related symptoms [18]. Testosterone replacement therapy was used in menopausal women to improve sexual desire, although it may have favourable effects on bones, muscles and cognitive function [19].

*Eurycoma longifolia* Jack (ELJ) is a small Asian tree belonging to the genus Eurycoma, commonly called as Tongkat Ali and Long Jack. The roots of ELJ are often called “Malaysian ginseng” [20] and is used for their anti-malaria, anti-cancer and anti-ulcer properties. It has also been commonly prescribed in traditional medicine as a febrifuge and a remedy for dysentery, glandular swelling and fever [21, 22]. ELJ has been reported to have antioxidative properties due to its high concentration of superoxide dismutase [19, 20]. ELJ supplementations were able to prevent the increase in bone resorption rate after orchiectomy by suppressing the elevation of C-terminal telopeptide of type I collagen [23].The root of ELJ is traditionally used globally for male sexual dysfunction and as an aphrodisiac. The compounds of the roots of this plant are scientifically tested and reported to have aphrodisiac and testosterone enhancing effects in the rat [24] as well as humans [25].

Hormone replacement therapy (HRT) is the strategy currently used for preventing and treating the symptoms of post menopause and post-menopausal related osteoporosis [26]. Ironically, long term HRT has been associated with increased risk of undesired side effects including headache, fluid retention, swollen breasts, breast cancer, endometrial cancer, venous thromboembolism, and cardiovascular disease [26, 27]. Consequently, there are no treatments that can be used safely in the long term in the management of post-menopausal syndromes, thus, it is necessary to develop a new drug of natural or synthetic origin, with minimal side effects [28].

The objective of the study was to evaluate if PEL is effective in maintaining hormonal balance and bone loss and to determine whether it is due to PEL’s ability to increase testosterone levels as testosterone has been used to manage menopausal symptoms. One of the treatment groups in the study received testosterone undecanoate 10 mg/kg every 4 weeks [19, 29] as a source of testosterone and this group served as positive control group. The no observed adverse effect level (NOAEL) of ELJ water extract (Physta®) was concluded as more than 1000 mg/kg orally based on acute, sub acute and 90 days sub-chronic studies [30]. As there was no study or any preliminary work available for ELJ on female reproductive hormone, a low 100 mg/kg, medium 300 mg/kg and high 500 mg/kg doses which well below the NOAEL were used in the study. In the present study, serum calcium, phosphate, bone alkaline phosphatase, ostoecalcin, follicle stimulating hormone (FSH), luteinizing hormone (LH), progesterone, testosterone, oestrogen levels and uterus body weight of OVX rats treated with PEL were evaluated to elucidate the promising effects in management of menopausal related symptoms.

## Methods

### Collection and preparation of plant extract

**Fig. 1:**
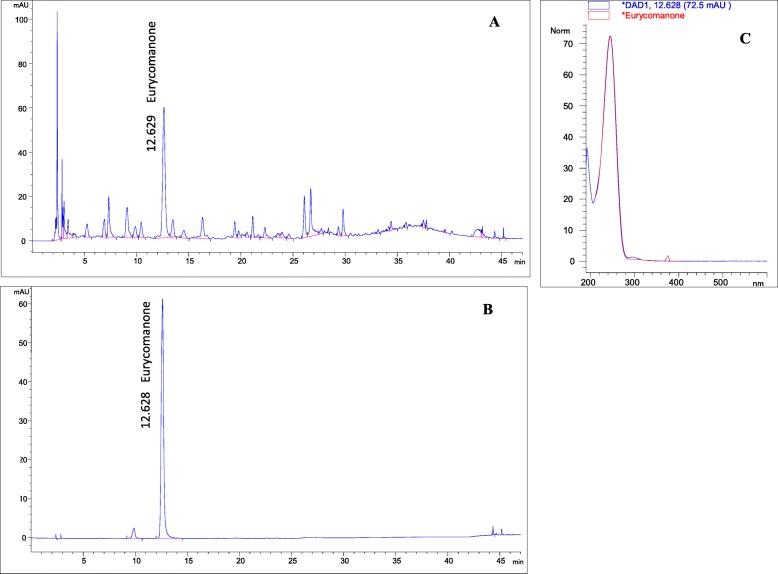
HPLC profile of PEL water extract. **a** The major peak corresponds to Eurycomanone at RT 12.629 min. **b** Eurycomanone standard eluted at RT 12.628 min. **c** UV spectrum of Eurycomanone with respect to its’retention time

ELJ extract used in the study was commercially available from Biotropics Malaysia under the trade name of Physta^®^, PEL extract from batch number TA 170750 was used in this study. It is a water extract of the roots of ELJ, standardised based on Malaysian Standard for ELJ water extract MS 24089:2011 [31], with specification of 0.8–1.5% eurycomanone, not less than 22% of total protein, not less than 30.0% of total polysaccharide and not less than 40.0% of glycosaponin. The HPLC fingerprint of PEL water extract was obtained according to the HPLC method using Kinetex 2.6 μm EVO C18 100 Å (150 × 4.6 mm) column. The mobile phase consisted of solvent A-0.02% trifluoroacetic acid in water and B - acetonitrile. A low gradient program with flow rate of 0.6 ml/min was set to t = 0 min 5% B; t = 9 min 7%B; t = 12 min 7.4% B; t = 15 min 8% B followed by isocratic of 12% B between t = 17–23 min and 18% B from t = 24–28 min. A gradual gradient was followed from t = 30 min 20%B; t = 35 min 30%B, t = 40 min 35%B before final t = 45–47 min at 95%B.The major peak in chromatogram was compared against Eurycomanone standard (Fig. 1).

### Experimental animals

The experimental protocol was approved by the Institutional Animal Ethics Committee (IAEC), proposal no. RR/IAEC/46–2017. The test facility, Radiant Research Services Pvt. Ltd. has been registered with *Committee for the Purpose of Control and Supervision of Experiments on Animals (CPCSEA), New Dehli for in-house breeding and experimentation purpose.* Forty-eight female in-house breed of 12-week old Sprague Dawley rats weighing 120–250 g were acclimatised in polypropylene cages with stainless steel top grill in standard laboratory conditions of 22 ± 3 °C, 30–70% relative humidity and a 12 h light-dark cycle. They were maintained under standard housing conditions with free excess to a standard diet (M/s. Amruth labs, Bangalore, India) and water ad libitum during the study. During acclimatization period 5 rats were housed in a single cage according to CPCSEA guideline and during dosing period (after surgical) single rat was placed in a cage. Sterile paddy husk was used as bedding material and changed at least twice a week.

**Fig. 2:**
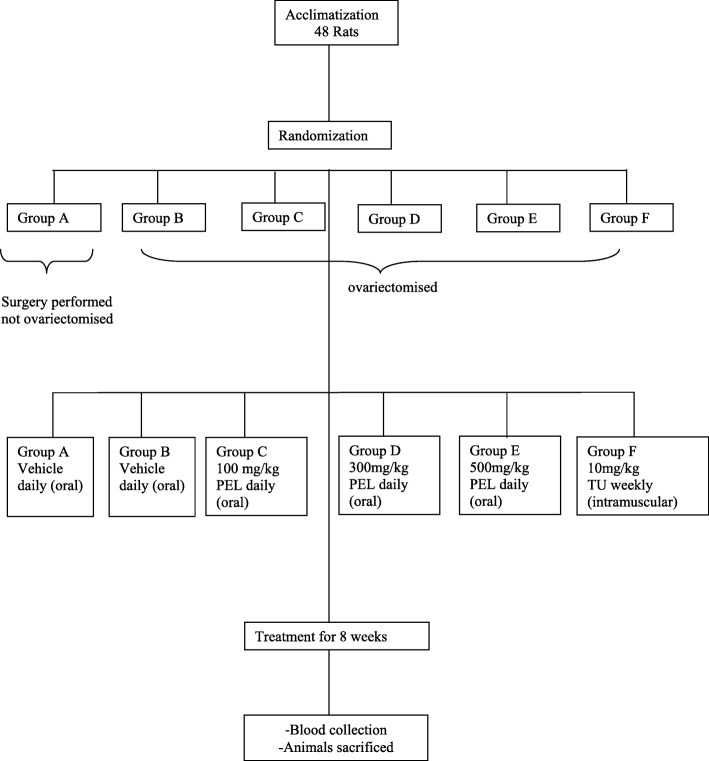
Randomization and Treatment Schedule Flow Chart

The animals were randomly divided into six experimental groups (Fig. 2), with each group consisting of eight rats. All animals were uniquely marked with picric acid and recorded. Group A: Sham operated control group (rats were operated on but the ovaries were not removed), received vehicle (0.5% carboxy methyl cellulose sodium) for 8 weeks; Group B: Untreated group (surgery was performed and ovaries were removed), received vehicle for 8 weeks; Group C: Low dose group, ovariectomised animals received daily oral treatment of PEL 100 mg/kg body weight for 8 weeks; Group D: Mid dose group, ovariectomised animals received daily oral treatment of PEL 300 mg/kg body weight for 8 weeks; Group E: High dose group, ovariectomised animals received daily oral treatment of PEL 500 mg/kg body weight for 8 weeks; Group F: Positive control group, ovariectomised animals receiving testosterone undecanoate (TU) (Sun Pharma Laboratories Ltd) 10 mg/kg body weight intramuscularly once in every weeks for 8 weeks. During the experimental period the animals were dosed during 10 to 11 a.m. Animals were dosed at the volume of 10 ml/kg based on most recent recorded body weight. Clinical signs, mortality and feed intake were determined daily, whereas body weight was recorded before surgery, weekly thereafter and at the end of the dosage schedule. All the procedures were carried out in procedure room.

At the end of the treatment period, the rats were fasted overnight, and 1.5 ml blood was collected via a retro orbital route. The rats were anaesthetised with 5% isoflurane the commonly used inhalant aesthetic [32]. The serum samples were prepared by centrifugation (3000 rpm for 10 mins) of the collected blood samples and then stored at − 80 °C for biochemical determinations. Animals were then sacrificed using 5% extended isoflurane anaesthesia (Raman & Well Pvt. Ltd) through inhalation the uterus was dissected out, adhering fat was removed, washed with saline solution and immediately weighed. After removing uterus those animals which gets recovered from the extended isoflurane anaesthesia was euthanized by CO_2_ inhalation method at a flow rate of 5 L/min (CO_2_ flow was maintained for at least 1 min after respiratory arrest).

### Surgical procedure

Anaesthesia was induced by intraperitoneal injection of ketamine HCl (80 mg/kg) (Neon Laboratories Ltd) as it has less effect on the rate and blood pressure. The depth of anaesthesia was checked by monitoring the respiratory rate or simply testing the animal response to gentle pressure on the hind paws. After the onset of anaesthesia, clippers were used to clip the skin and fur shaved bilaterally to expose the skin. The procedure was conducted by making two incisions to separately reach each ovary. The shaved skin was swab with 70% (v/v) ethanol and the anaesthetised animal was placed on the operating table with its back exposed and its tail towards the operator. A single dorsal incision (1 cm) was made penetrating the skin using small scissors in the lower back, directly below the bottom of the rib cage. Gently subcutaneous connective tissue was made freed from the underlying muscle on each side using blunt forceps. The ovary was located under the thin muscle layer and a small incision (less than 1 cm) was made on each side to gain entry to the peritoneal cavity. The edge of the incision was held securely with tooth forceps and the ovarian fat pad was retracted surrounding ovaries with blunt forceps to expose the oviduct. The ovary was removed by gently severing the oviduct, using sterile, small scissors. The uterus and remaining part of the oviduct was replaced back into the abdominal cavity. The muscle layer was sutured and the procedure was repeated for the other ovary. A suitable analgesic meloxicam (Intas Pharmaceuticals Ltd) was administered subcutaneously at a dose of 5 mg/kg body weight post operatively [32].

### Biochemical analysis

Serum concentrations of calcium, phosphate, bone alkaline phosphatase, ostoecalcin, FSH, LH, progesterone, testosterone and oestrogen were measured using commercial assay kits. Calcium and phosphate levels were measured using a fully Automated Clinical Chemistry Analyzer EM360, Transasia Bio-medicals Ltd. Calcium was analysed by an ERBA calcium kit (Lot No: B071648) by the method of aresenazo III. Phosphate was analysed using the ERBA Phosphate kit (Lot No: B071630) by an ammonium molybdate method using a biochemistry EM 360 analyser. Bone type of alkaline phosphatase was assayed using an Elabsciences ELISA kit (Cat No.: E-EL-R1109). Osteocalcin was tested using Elabsciences ELISA kit (Cat No.: E-EL-R0243). FSH was analysed using a Roche Follicle Stimulating Hormone kit (Lot No: 033208), LH was analysed using a Roche Luteinising Hormone kit (Lot No: 030208). Oestrogen was analysed using a Roche Estradiol kit (Lot No: 127038), Progesterone was analysed by a Roche progesterone kit (Lot No: 0278) and testosterone was tested using a Roche Testosterone kit (Lot No: 180003). All these reproductive hormone estimations were performed by the method of C4SA (CLIA Chemiluminescence immunoassay) using a Siemens fully auto analyser (Model No. EXL-200) according to the manufacturer’s protocol.

### Statistical analysis

All data including body weight, feed consumption, biochemical evaluation and organ weights were statistically analysed using Graph-Pad Prism Software, version 5.01. All values were expressed as mean ± SEM. The significant difference between the treatment and control group was estimated using one-way ANOVA with Tukey’s post test and the results were considered statistically significant at *P* < 0.05.

## Results

### Effect on body weight of rat

The rats in all experimental groups had similar initial body weights, but 8 weeks after surgery, the untreated rats showed a significant increase in body weight gain (41.22 ± 2.85%, ^##^*P* < 0.01) compared to the control group. Treatment with PEL 500 mg/kg prevented the ovariectomy-induced weight gain (36.00 ± 1.44%) when compared to the untreated group (41.22 ± 2.85%) at week 8, with the sham control group showing the lowest percentage weight gain (29.86 ± 1.98%) at week 8. The increase in body weight from the start of the study to 8 weeks was inhibited in PEL treated groups in a dose dependent manner. No significant changes were observed in the percentage weight gain in all treatment groups when compared to the untreated group during the study period (Table 1).

**Table 1:** Effect of PEL on percentage (%) rat body weight gain

Groups	Weight (g)		Percentage Weight Gain (%) Week 8
	Basal	Week 8	
Group A Sham control	186.8 ± 9.60	242.1 ± 7.62	29.86 ± 1.98
Group B Untreated	185.1 ± 5.45	261.1 ± 7.73	41.22 ± 2.85^##^
Group C PEL 100 mg/kg	185.4 ± 5.32	258.5 ± 6.82	39.59 ± 1.66
Group D PEL 300 mg/kg	185.4 ± 6.28	253.9 ± 7.20	37.41 ± 3.31
Group E PEL 500 mg/kg	185.8 ± 6.23	252.5 ± 8.31	36.00 ± 1.44
Group F Positive control	185.0 ± 3.35	259.3 ± 4.85	40.14 ± 0.73

Data stated as mean ± SEM; ^##^*P* < 0.01 vs. Group A

### Effect on organ weight

Ovariectomy resulted in a significant reduction (*P* < 0.001, 238.81 ± 5.39 mg) in uterus weight in the ovariectomised rats and administration of PEL and testosterone undecanoate after ovariectomy inhibited further loss of uterus weight. The administration of testosterone showed a significant increase (*P* < 0.001, 389.74 ± 8.26 mg) in uterine weight compared to the untreated group. An increasing dose-dependent trend was observed in PEL treatment groups (Table 2).

**Table 2:** Effect of PEL on uterus weight

UTERUS WEIGHT (mg)					
Group A	Group B	Group C	Group D	Group E	Group F
679.06 ± 6.65	238.81 ± 5.39^###^	235.84 ± 4.03	240.73 ± 2.93	256.39 ± 4.41	389.74 ± 8.26^***^

Data stated as mean ± SEM; ^###^ *P* < 0.001 vs. Group A; ***P < 0.001 vs. Group B

### Effect on serum biochemistry

The effects of PEL on serum biochemical markers are summarised in Tables 3 and 4. Untreated rats showed a significant decrease in calcium (*P* < 0.001, 7.86 ± 0.12 mg/dl), phosphate (*P* < 0.001, 2.83 ± 0.12 mg/dl) and bone specific alkaline phosphatase (BALP) (*P* < 0.001, 2.54 ± 0.20 ng/ml) compared to the sham control group. However, in the positive control group animals treated with testosterone undecanoate, there was a significant increase in calcium (*P* < 0.05, 8.48 ± 0.16 mg/dl), phosphate (*P* < 0.05, 3.29 ± 0.13 mg/dl) and BALP (*P* < 0.05, 3.06 ± 0.12 ng/ml) levels compared to the untreated group. Similarly, treatment with PEL increased the serum calcium, phosphate and BALP levels in a dose dependent manner compared to the untreated group. There were no significant changes observed in the ostoecalcin levels in all the treated groups compared to the untreated group. Ovariectomy appears to significantly increase serum FSH (*P* < 0.001, 5.70 ± 0.33 mIU/ml) and LH (*P* < 0.001, 4.69 ± 0.22 mIU/ml) levels in the untreated group compared to the sham control group. However, treatment of ovariectomised rats with TU significantly reduced the serum FSH (*P* < 0.001, 3.96 ± 0.22 mIU/ml) and LH (*P* < 0.001, 3.76 ± 0.11 mIU/ml) levels compared to the untreated group. The effect of PEL 500 mg/kg on serum FSH was significant, where it was reduced compared to the untreated group (*P* < 0.001, 4.25 ± 0.22 mIU/ml), but the decrease in the level of serum LH did not reach statistical significance. A significant drop in serum levels of progesterone, oestrogen and testosterone were observed in the untreated group compared to the sham control group, whereas there was a significant increase in the levels of progesterone (*P* < 0.001, 3.31 ± 0.15 ng/ml), oestrogen (*P* < 0.001, 12.35 ± 0.25 pg/ml) and testosterone (*P* < 0.001, 13.01 ± 0.41 ng/ml) in the positive control group. As expected, progesterone (*P* < 0.05, 2.48 ± 0.08 ng/ml), oestrogen (*P* < 0.05, 11.02 ± 0.13 pg/ml) and testosterone (NS, 1.83 ± 0.03 ng/ml) levels were increased by treatment with PEL 500 mg/kg (Table 3).

**Table 3:** Effect of PEL on serum calcium, phosphate, BALP and osteocalcin levels

Groups	Calcium (mg/dl)	Phosphate (mg/dl)	Bone Alkaline phosphatase (ng/ml)	Osteocalcin (ng/ml)
Group A Sham control	9.05 ± 0.20	3.71 ± 0.14	3.43 ± 0.11	7.13 ± 0.34
Group B Untreated	7.86 ± 0.12^###^	2.83 ± 0.12^###^	2.54 ± 0.20^###^	7.86 ± 0.48
Group C 100 mg/kg	7.85 ± 0.12	2.83 ± 0.11	2.57 ± 0.04	7.89 ± 0.06
Group D 300 mg/kg	7.98 ± 0.11	2.89 ± 0.08	2.70 ± 0.11	7.79 ± 0.21
Group E 500 mg/kg	8.11 ± 0.11	3.01 ± 0.12	2.82 ± 0.10	7.52 ± 0.18
Group F Positive control	8.48 ± 0.16*	3.29 ± 0.13*	3.06 ± 0.12*	7.26 ± 0.20

Data stated as mean ± SEM; ^##^*P* < 0.05, ^##^*P* < 0.01 and ^###^*P* < 0.001 vs. Group A; *P < 0.05, **P < 0.01 and ***P < 0.001 vs. Group B

**Table 4:** Effect of PEL on serum FSH, LH, progesterone, oestrogen and testosterone levels

Groups	FSH (mIU/ml)	LH (mIU/ml)	Progesterone (ng/ml)	Oestrogen (pg/ml)	Testosterone (ng/ml)
Group A Sham control	2.26 ± 0.07	1.73 ± 0.05	5.78 ± 0.25	29.31 ± 0.60	4.48 ± 0.21
Group B Untreated	5.70 ± 0.33^###^	4.69 ± 0.22^###^	1.82 ± 0.09^###^	9.49 ± 0.27^###^	1.22 ± 0.06^###^
Group C PEL 100 mg/kg	5.29 ± 0.27	4.57 ± 0.18	1.88 ± 0.05	9.52 ± 0.15	1.21 ± 0.04
Group D PEL 300 mg/kg	4.87 ± 0.20	4.36 ± 0.20	1.96 ± 0.09	10.18 ± 0.28	1.31 ± 0.03
Group E PEL 500 mg/kg	4.25 ± 0.22^***^	4.07 ± 0.12	2.48 ± 0.08^*^	11.02 ± 0.13^*^	1.83 ± 0.03
Group F Positive control	3.96 ± 0.22^***^	3.76 ± 0.11^**^	3.31 ± 0.15^***^	12.35 ± 0.25^***^	13.01 ± 0.41^***^

Data stated as mean ± SEM; ^#^*P* < 0.05, ^##^*P* < 0.01 and ^###^*P* < 0.001 vs. Group A; *P < 0.05, **P < 0.01 and ***P < 0.001 vs. Group B

## Discussion

Ovariectomy is a standard surgical procedure to induce menopause in experimental animals and ovariectomised female rats show a dramatic cessation of ovarian function and higher risk of osteoporosis [33, 34]. The effect of ovariectomy was clearly seen in the untreated group where level of investigated hormones, serum bone biomarkers, body weight gain and uterus weight were significantly altered compared to the sham control group. The study findings show supplementation of PEL and testosterone alleviated the changes in all the parameters investigated in ovariectomised rats.

The weight gain in ovariectomised rats was significantly higher compared to the sham control group, which may be related to oestrogen insufficiency. The effect of oestrogen insufficiency on lipid metabolism during menopause has been well documented [34], and it is the main reason for an increase in adiposity, particularly abdominal fat accumulation [34]. Even though not significant, supplementation of PEL has ability to inhibit weight gain in a dose dependent manner.

The ovariectomised rats that did not undergo any treatment showed a significant decrease in uterine weight compared to those rats which only underwent a sham operation. The reduction of uterine weight was due to an atrophy of endometrium resulting from a lack of hormones secreted by the ovaries [34]. Oestrogen plays a predominant role in reducing uterine weight gain. The ovariectomy caused reduction in oestrogen hormone, thereby reducing uterine oestrogen receptors, leading to a decrease in the proliferative layers, luminal epithelium, thin stroma and myometrium, subsequently reducing uterus weight [35]. However, the administration of PEL to ovariectomised rats for 8 weeks appeared to reduce the weight loss, mainly by minimising uterine atrophy compared to the untreated ovariectomised rats. This improvement in uterine atrophy and weight is probably due to increases in the oestrogen levels in PEL supplemented groups.

The prevalence of osteoporosis increases with age, whereby bone loss (loss of calcium, phosphate and BALP) is reportedly more rapid in females in the first few years post menopause and is influenced by oestrogen deficiency [36]. In the present study, ovariectomy caused a significant loss of calcium and phosphate levels in untreated rats when compared to the sham control rats, similar to other published studies [37, 38]. This could be related with ovarian hormone deficiency inducing the synthesis of cytokines by osteoblasts, monocytes and T cells, thereby initiating bone resorption by increasing osteoclast activity [39]. The action cause reduced intestinal calcium absorption and may contribute towards a lower level of calcium in blood serum. A pre-clinical study on rats supports the positive effects of TA on bone turnover in androgen-deficient rats [40], as TA supplementation reduced the percentage of osteoclasts and increased the percentage of osteoblasts on the bone surface [40]. The measurement of BALP activity can be used as a marker of bone formation and bone resorption in vivo [34, 41]. Treatment with PEL has the potential to reduce bone loss in the OVX rats by elevating calcium, phosphate and BALP levels.

The onset of menopause is associated with a dramatic change in hormonal balance, a decrease in oestrogen and increase in FSH and LH hormones, which ultimately reduces the level of progesterone and causes permanent amenorrhea [42, 43]. Accordingly, serum FSH and LH levels were significantly increased in the untreated group compared to the sham control group, possibly due to the increased release of luteinising hormone releasing hormone which has been shown to cause higher serum FSH and LH levels in menopausal female rats [35]. These biomarkers were significantly decreased in OVX rats treated with PEL compared to the untreated rats. A significant depletion in the level of progesterone, oestrogen and testosterone in the untreated group provides evidence that the ovariectomy results in post-menopausal like symptoms. Administration of PEL at high dose to the OVX rats significantly increased the levels of progesterone and oestrogen, and there was a trend for increased testosterone level. This corroborates with a previous study in which ELJ increased testosterone levels in hypogonadic men [25], hence has the ability to trigger the production of testosterone from other organs such as adrenal cortex. There are few mechanisms to explain the increase in testosterone by ELJ supplementation. High performance liquid choromatography (HPLC) analysis of aqueous extract and fractions from various ratio of water-methanol extraction shows that ELJ contain four major quassinoids ie. eurycomanone, 13α(21)-epoxyeurycomanone, 13,21-dihydroeurycomanone and eurycomanol [44]. The effect of these quassinoids on male fertility and testosterone production was investigated and found to increase testosterone levels in the testis and plasma of rats supplemented with eurycomanone fraction. The same study also concluded that the testosterone elevating effect of eurycomanone was dose dependant. As such it is possible that eurycomanone could be one of the bioactive markers of PEL.

Apart from eurycomanone ELJ is reported to contain peptide which enhance the biosynthesis of various androgens [45]. The peptide was shown to activate the CYP17 (17 α-hyroxylase/17, 20 lyase) enzyme to enhance the metabolism of pregnenolone and 17-OH-pregnenolone to yield more dehyroepiandrosterone (DHEA). Progesterone and 17-OH-progesterone are further metabolised to 4-androstenedione and testosterone [46]. As testosterone is the precursor hormone for oestrogen via aromatisation, the testosterone can be further aromatised to oestrogen. This explains the increase in the oestrogen levels in animals treated with PEL containing the bioactive peptide.

Most menopausal symptoms are caused by fluctuating hormone levels. Supplementation of PEL can stabilise the fluctuation of hormone levels in the ovariectomised rats, indicating the potential of PEL to alleviate menopausal related symptoms. Future investigations will evaluate other parameters, such as bone mineral density, additional bone formation markers, bone resorption markers, histology of uterus and tibia bone to further understand how PEL can minimise changes in hormonal and bone related biomarkers in ovariectomised rats.

## Conclusion

The present study, demonstrated that PEL could reduce bone loss and improve hormonal levels caused by ovariectomy, an animal model depicting menopause. Furthermore, the effects of PEL on reproductive hormones and bone markers were dose dependent. Hence, PEL may be considered for menopause management and clinically evaluated in future studies in this area.

## Data Availability

The datasets used and/or analysed during the current study available from the corresponding author on reasonable request.

## Abbreviations

ALP: Alkaline phosphatase
ANOVA: One-way analysis of variance
BALP: Bone specific alkaline phosphate (BALP)
BFM: Bone formation markers
bw: Body weight
CLIA: Chemiluminescence immunoassay
CPCSEA: Committee for the Purpose of Control and Supervision of Experiments on Animals
CTx: C-terminal telopeptide of type I collagen
DHEA: Dehydroepiandrosterone
ELISA: Enzyme linked immune sorbent assay
ELISA: Enzyme linked immunoassay kit
ELJ: *Eurycoma longifolia* Jack
FSH: Follicle stimulating hormone
HCL: Hydrochloric acid
HRT: Hormone replacement therapy
IAEC: Institutional Animal Ethics Committee
LH: Luteinising hormone
LHRH: Luteinising hormone releasing hormone
OVX: Ovariectomy
PEL: *Physta* ^®^ *Eurycoma longifolia*
TU: Testosterone undecanoate
